# NADA Protocol for Behavioral Health. Putting Tools in the Hands of Behavioral Health Providers: The Case for Auricular Detoxification Specialists

**DOI:** 10.3390/medicines5010020

**Published:** 2018-02-07

**Authors:** Elizabeth B Stuyt, Claudia A Voyles, Sara Bursac

**Affiliations:** 1 Department of Psychiatry, University of Colorado Health Sciences Center, Pueblo, CO 81007, USA; 2 Department of Clinical Studies, AOMA Graduate School of Integrative Medicine, Austin, TX 78745, USA; claudiavoyles@yahoo.com; 3 National Acupuncture Detoxification Association, Laramie, WY 82070, USA; sarabursac@gmail.com

**Keywords:** NADA: National Acupuncture Detoxification Association, NADA protocol, acudetox, ADS: Auricular (or Acupuncture) Detoxification Specialist

## Abstract

**Background:** The National Acupuncture Detoxification Association (NADA) protocol, a simple standardized auricular treatment has the potential to provide vast public health relief on issues currently challenging our world. This includes but is not limited to addiction, such as the opioid epidemic, but also encompasses mental health, trauma, PTSD, chronic stress, and the symptoms associated with these conditions. Simple accessible tools that improve outcomes can make profound differences. We assert that the NADA protocol can have greatest impact when broadly applied by behavioral health professionals, Auricular Detoxification Specialists (ADSes). **Methods:** The concept of ADS is described and how current laws vary from state to state. Using available national data, a survey of practitioners in three selected states with vastly different laws regarding ADSes, and interviews of publicly funded programs which are successfully incorporating the NADA protocol, we consider possible effects of ADS-friendly conditions. **Results:** Data presented supports the idea that conditions conducive to ADS practice lead to greater implementation. Program interviews reflect settings in which adding ADSes can in turn lead to improved outcomes. **Discussion:** The primary purpose of non-acupuncturist ADSes is to expand the access of this simple but effective treatment to all who are suffering from addictions, stress, or trauma and to allow programs to incorporate acupuncture in the form of the NADA protocol at minimal cost, when and where it is needed. States that have changed laws to allow ADS practice for this standardized ear acupuncture protocol have seen increased access to this treatment, benefiting both patients and the programs.

## 1. Introduction

**Figure 1:**
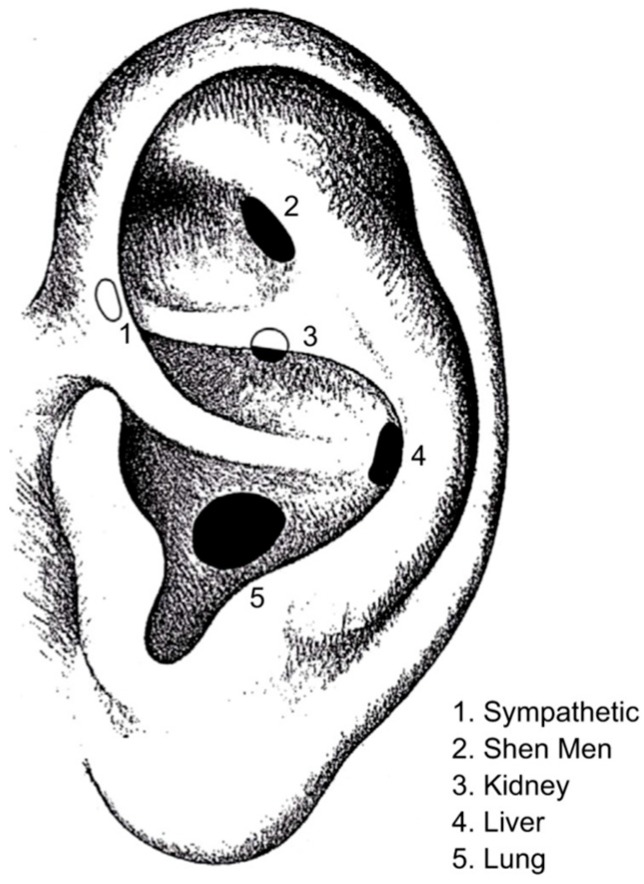
National Acupuncture Detoxification Association (NADA) Protocol. The points: 1 Sympathetic; 2 Shen Men; 3 Kidney; 4 Liver; and 5 Lung provide balance and yin nourishment with the presumptive diagnosis of yin deficiency, “empty fire”, and the conventional medicine diagnoses of behavioral health. The NADA protocol includes bilateral manual needling of one to five points typically delivered frequently (often daily) with participants sitting quietly in groups for 30–45 min, or the application of seeds/beads, often just on Shen Men or Reverse Shen Men (opposite Shen Men on the back of the ear).

The National Acupuncture Detoxification Association (NADA) protocol, also known as acudetox, originally designed for use in acute heroin withdrawal, has become a remarkably effective tool applied to many populations receiving behavioral healthcare and primary healthcare [1]. Not a replacement for other modalities, the NADA protocol is always understood to be best utilized in an integrated setting with the right match of bio/psycho/social/spiritual supports and interventions. Nor is the NADA protocol a replacement for Chinese medical care which excels at diagnosis and individualized treatment, including a broader range of auricular interventions (see Figure 1). 

Integrated Behavioral Health Care is where behavioral health and medical providers work together to help address the emotional, mental, and spiritual side of any medical condition in order to improve outcomes [2]. The factors we call stress or stressors contribute to worse outcomes in healthcare whether in primary care, or in illnesses more traditionally understood as behavioral health disorders: mental illness, stress, grief and loss, addiction, trauma, and disaster responses. 

The NADA protocol is a tool that can be broadly, efficiently applied by behavioral health professionals in a myriad of settings. What prevents or limits that happening has to do with access and availability of trained healthcare providers who can incorporate the protocol into existing systems with existing staff rather than relying on outside acupuncturists or medical acupuncturists. The ability of trained healthcare specialists to practice the protocol, as Auricular Detoxification Specialists (ADS), depends upon legislation, regulation, and scope definition. This paper makes the case for ADS provision of acudetox, reviewing the historical background and development of the NADA protocol and related legislation, exploring how legislation affects application and availability and offering examples of how the NADA protocol can flourish when appropriate measures allow that growth. 

### History and Background

The NADA protocol was developed in the mid-1970s at Lincoln Detox through a community process of experimentation and feedback [3]. From the research of Chinese neuro-surgeon, H.L. Wen, the Lincoln Detox group had learned about the effects of the ear lung point (with electrostimulation) in relieving acute opium withdrawal symptoms [4]. From there, the protocol developed into what is now known as the NADA protocol. 

The people administering the NADA protocol at Lincoln Detox were a variety of frontline staff members—counselors, nurses, and peer recovery workers. Although formal acupuncture education was in its early stages, it was still not legal practice to have acupuncture needles inserted into the body by non-physicians [3]. The grassroots application of the NADA protocol at Lincoln even caused several brief closures of the program. 

Considering the benefit that clients experienced with the ear acupuncture treatment, advocates formed the NADA organization in 1985 to facilitate its growth. In 1989, Lincoln Detox’s medical director, Michael O. Smith, helped petition for the first law in the United States allowing non-acupuncture personnel to provide this standardized and limited protocol. The new law stipulated that individuals could be Acupuncture Detoxification Specialists (ADSes) as long as they worked in a setting that also provided comprehensive addiction treatment services. It also required them to be supervised by a licensed acupuncturist or a physician with acupuncture training.

Lincoln Detox, renamed Lincoln Recovery Center in the early 1990s, became a thriving training center for ADSes, both from within and outside of the United States. The two-week training program was apprenticeship-based and would welcome a new group of trainees each week. By always having a group of trainees in the clinic room, Smith did not need to hire additional staff to provide the treatment. In this manner they were able to keep the training free of charge, and thus very accessible to many New York-based programs. 

When those trainees returned to their programs, they provided the treatment as an addition to the existing array of services. Word spread about the innovative training and clinical services pioneered by Lincoln Recovery Center, through published research, congressional hearings, national conferences, and invited talks given by Michael Smith around the country. Gradually the practice of acudetox spread. The NADA organization estimates that some twenty-five thousand persons have been trained in this method worldwide [5]. 

The intervention is inexpensive and easily adopted especially when it can be provided by behavioral health treatment professionals either individually or within an integrated system of care. The practice of training behavioral health providers as ADSes is allowed by some but not all of the states in the U.S., and some but not all of the countries in the world [5]. To date there are twenty-one (21) states that have a statute giving a diverse group of healthcare workers the ability to be trained in the NADA protocol [6]. U.S. state laws vary in terms of who can perform the protocol, whether their scope is behavioral health or just addiction, where they can practice, and the kind of training, supervision, and oversight required. 

The preponderance of clinical/anecdotal and evidence-based experience indicates that the NADA model of care improves treatment and health outcomes. The model includes the following components: (1) integration within other interventions; (2) barrier-free; (3) regular treatments; (4) a communal setting; and (5) local personnel and/or cross-trained health providers offer the therapy [7].

In 1993, according to a government survey of public and private substance abuse treatment facilities, there were 57 New York state programs reporting the use of acupuncture [8]. By 2000, that number had more than quadrupled, growing to 234. (The Substance Abuse, Mental Health Services Administration (SAMHSA) conducts an annual survey of addiction treatment programs and since 1992, that survey has included acupuncture amongst the “ancillary services”. Note that the survey question is about “acupuncture”, not auricular acupuncture or acudetox, and therefore does not distinguish the type of needling provided.). 

One of these reporting sites was the SISTERS program—Sustained Interpersonal Strategies for Treatment and Empowerment of Recovering Substance Abusers—located within the Lincoln Recovery Center’s Maternal Substance Abuse Services (MSAS) [9]. A peer counseling model, SISTERS operated from 1991 until 1996 with grant funding from the Center for Substance Abuse Prevention. In addition to other services, SISTERS staff would administer daily NADA treatments until the client could provide ten consecutive days of negative urine toxicologies. Women who participated in the SISTERS program had babies born with higher birth weights as compared to the national birth weight average for babies born to women in recovery, and 78% of the babies were born with negative toxicology tests at the time of delivery. Program participants also had greater rates of family reunification with children either not living with them or in the foster care system. 

The key takeaway from this program profile is that the services were run by peers who had themselves been successful graduates of the MSAS program. They coordinated services for SISTERS clients as well as provided the daily NADA acupuncture treatments. MSAS and the SISTERS program received national attention in the 1994 National Public Health and Hospital Institute publication, Vulnerable Women and Visionary Programs, which provided a review of programs that successfully helped drug-involved women and their children [10]. The publication notes that the effectiveness of peer counselors in improving outcomes was a key component.

A comparative study between two inpatient treatment programs, the Kent-Sussex Detoxification Center in Delaware which employed trained nurses as ADSes and an acudetox program at a Maryland hospital which hired acupuncturists, demonstrated a stark contrast in NADA service delivery. Study outcomes showed the use of ADSes in the Delaware program resulted in: (1) greater trust and rapport with clients; (2) treatment on demand—“with retention as the primary goal, the value of this service is inestimable”; (3) no additional cost to the program, save for the supplies—“Fees were incurred for consulting and the training of the nurses, but those fees were the equivalent of three weeks of acupuncture provided by licensed acupuncturists at the mental health hospital”, (4) administrative simplicity—“Organizing a group of acupuncturists and developing a schedule which takes time out of from their busy practices can be complex … only one face-to-face meeting with the five acupuncturists has been arranged in the six months”; (5) availability of acupuncturists—“Established acupuncturists are often unwilling or reluctant to interrupt their daily treatment schedules to travel fifteen to thirty minutes to a site where they earn considerably less”; (6) improved morale for the staff—“The improvement in the quality and efficacy of the program has come from internal resources”; and (7) staff become more potent agents for change—“The gift that acupuncturists can give to the field is the transfer of their knowledge and skills to those already working in the field [11] (pp. 11–12)”.

A Texas-based NADA trainer conducted a survey in 1996 to find out how willing and able full-body acupuncturists would be to provide their services, specifically the NADA protocol, in the public health sector [12]. The survey was administered to 233 licensed acupuncturists, and 60 expressed an interest. However, the majority of the interested acupuncturists had limited availability (one to two days per week) and expected to receive compensation. According to a program administrator quoted in the Guidepoints report at the time, “Our patients really look forward to the acupuncture and they are accustomed to having it available every day from their regular counselors. It doesn’t work well otherwise [12] (pp. 2–3)”.

A similar survey was administered to acupuncturists in Maine in 2017, although its scope was more general to public health, including addiction and mental health [13]. The survey results showed trends similar to the responses in the Texas study. A majority of acupuncturists responded that they did not want to join the new Maine Acupuncture Public Health and Wellness Committee to address the opioid epidemic in their state and would not be interested in volunteering either weekly, once a month or as a fill-in at one of four free acupuncture veterans clinics due to time constraints and travel distance. 53% responded that they offer pro bono acupuncture, but mostly out of their own clinic setting. Interestingly, 69% reported past experience working with clients and in programs treating addiction. However, most provided fairly limited timeframes that they could offer treatment to that demographic at present.

The west coast of the United States, primarily Oregon and California, have historically had many programs with hired acupuncturists providing the NADA protocol. A 2012 report revealed that due to budget cuts in the addiction treatment field, paid positions for acupuncturists in those states utilizing the NADA protocol sharply declined [14]. Neither of these states currently permits non-acupuncture ADSes.

While the research base for the NADA protocol has been mixed and does not include large scale replicated RCT trials, there is a growing body of positive small usual-care controlled studies and a large base of anecdotal reports [1]. One such study of NADA added to usual care in a residential substance abuse treatment program demonstrated statistically significant decreases in symptom severity [15]. Carter and his co-authors studied common symptoms associated with behavioral health disorders: mental/emotional (depression, anxiety, anger, concentration), and physical (cravings, decreased energy, and pain in the form of head and body aches). Two systematic reviews/meta-analysis studies address the benefits of auricular acupuncture and acupressure for pain [16,17]. The latter, which focused on emergency settings, includes studies of “battlefield acupuncture”, a standardized five-point protocol and style of auriculotherapy for treating acute pain widely adopted in military settings and applied by trained, non-acupuncturist, medical providers. 

A Kaiser Permanente HMO-based study demonstrates not only benefits of adding NADA-style treatment to usual addiction care, but also the cost effectiveness. In their evaluation of 44 patients, those who received NADA supported treatment were more successful. Usual care with acudetox added was both more effective and less expensive to deliver [18]. Looking more deeply into the text, the authors show that the first year start-up costs were inflated by the necessary training fees which would not be required in subsequent years, indicating that the cost savings would be even more pronounced over time. Furthermore, the program was in California and the needling providers were licensed acupuncturists, not the agency’s clinical staff. Using ADSes (which California law does not currently support) would render the cost savings even more significant. 

Treatment administrators have long known that even if they are not able to get direct reimbursement for the treatment itself, implementing the NADA protocol results in better outcomes, decreases clients leaving against advice, improves staff/client relationships and satisfaction, can be offered as staff wellness benefit, and improves marketing and competitive edge thereby paying for itself many times over.

While initially the NADA protocol was used as a supportive component in addiction treatment programs, after the 11 September 2001 attacks in New York City the protocol was discovered to be useful for people experiencing a severe traumatic event [19]. Its non-verbal nature helped people relax and sleep better which ultimately helped them feel better able to cope with the traumatic experience. The protocol was again used effectively in 2005 after hurricanes Katrina and Rita. That experience resulted in the passage of an ADS law in Louisiana—to increase access to this treatment tool for both addiction treatment and future disaster response capability. Another outgrowth of this NADA response was the founding of the nonprofit, Acupuncturists Without Borders [20]. This group now primarily relies on the NADA protocol to aid in disaster situations throughout the world. 

When laws and conditions in a given state are supportive of ADS practice, the availability of the NADA protocol will increase. However, when the state law is not supportive of non-acupuncturist ADS practice, there is less provision of the NADA protocol and therefore less public benefit. We have described some historical evidence of that and present the current situation in the United States. 

## 2. Materials and Methods

In order to compare states with varying legislative landscapes, we employed several qualitative methods: (1) conducted a survey of NADA providers within three representative states; (2) compiled information from available databases and sources; and (3) conducted brief interviews of providers in a sample of programs in Colorado.

(1) Survey: A simple two-question email survey was sent to all NADA-trained persons (740) in the following states: California (CA), Colorado (CO), and Georgia (GA), chosen as representative of the range of ADS practice. 1-CA, a state with many licensed acupuncturists and no ADS statute, 2-CO, a state with a recent and more flexible ADS law with no supervision requirement, 3-GA, a state that has an ADS law but requires direct supervision by an acupuncturist.
(2) Search and compilation of state data: The U.S. government, through SAMHSA, conducts the annual National Survey of Substance Abuse Treatment Services (N-SSATS) as mentioned above. This information provides a snapshot of the substance abuse services being used in the public/private addiction treatment sector. Specifically, we looked at SAMHSA’s 2016 data which we combined with the NADA database of trained NADA providers who are active members of NADA, and state-reported data about licensed acupuncturists.

In addition, we ranked states based upon their ADS legislation, taking into account scope and supervision factors. While state laws vary significantly, we have grouped all 50 states into three categories. Category 1 indicates “ADS-friendly” states where a statute allows various groups to perform the NADA protocol either without supervision or under general supervision and with broad behavioral health applications/scope of practice. Category 2 is also states with statutory privileges for some groups to perform the NADA protocol. These states, however, have more restrictive supervision requirements or significant limits on the setting or population. Category 3 includes three types of states: those where only acupuncturists and/or physicians can perform the protocol and those in which the ADS regulations are restrictive to the degree that they deter any real practice of the NADA protocol. Lastly, this group includes states with no acupuncture regulation.

(3) To illustrate the type of implementation that can occur with supportive legislative changes, we conducted brief interviews with programs which have added acudetox into integrated health settings.

## 3. Results

### 3.1. Three-State Survey

We emailed 740 surveys to all NADA-trained persons in the organization’s database, with the following response rate: California 33/295, 11.2%; Georgia 10/47, 21.3%; Colorado 92/398, 23.1%. The respondents reported on their knowledge of acudetox-using programs in their state as follows: California 34; Georgia 5; and Colorado 70. 

See Figure 2.

### 3.2. Compilation of State-by-State Data 

Using the SAMHSA data and information from each state regarding the number of licensed acupuncturists and ADSes, a state-by-state comparison was made including the number of licensed acupuncturists as reported by state-based regulatory authorities, the number of substance abuse treatment programs, the percent of treatment programs reporting the use of acupuncture and the number of ADSes with an active membership in NADA. States reporting the highest number of acupuncture services within substance abuse programs also have ADS-friendly laws. (By this ranking system, there are five states in category one, 19 in two, and 26 in three). See Table 1. 

Comparing the results obtained from the survey of providers in the three representative states and the data obtained from SAMHSA there are some evident discrepancies. While the numbers for Georgia are consistent for both surveys—only five programs in that state and only 1.6% of the total programs report using acupuncture, practitioners in Colorado report more programs using acudetox, 70, than obtained in the SAMHSA survey, 39, which may reflect the broader inclusion in behavioral health and integrated health programs and not just substance abuse treatment programs. The discrepancy between the data for California—survey of NADA trained providers indicating 34 programs currently using acupuncture and the SAMHSA survey indicating 115 using acupuncture may be reflective of private programs utilizing full-body acupuncture, not just the NADA protocol. At the same time the 115 are only 8% of the total 1430 substance abuse treatment programs in California that report using acupuncture as an ancillary service. 

### 3.3. Program Profiles Based on Personal Interviews

In Colorado, a law was passed in 2013 allowing for NADA training of licensed individuals in the behavioral health care field, including Licensed Clinical Social Worker (LCSW), Licensed Addiction Counselor (LAC), Licensed Professional Counselor (LPC), Licensed Marriage and Family Therapist (LMFT), Certified Addiction Counselor III (CAC III), and licensed psychologists. These professional groups were granted an allowance to utilize the NADA protocol in their respective practices without supervision, after successfully completing the NADA training (these clinicians have a license which allows them independence as mental health practitioners in the state of Colorado). 

The NADA protocol is a safe procedure with minimal side effects [21]. The training includes clean needle technique and ADS providers have demonstrated knowledge and competence to continue to use this procedure safely and effectively without ongoing supervision. In the four years since the CO law has gone into effect, there has been a significant expansion of the availability of this treatment statewide. It is being implemented in private and publicly-funded substance abuse treatment programs, inpatient and outpatient mental health facilities, as well as fully integrated physical and behavioral health programs. Exposure to the NADA protocol has also increased the interest in acupuncture in general, with more people reporting an interest to the authors in pursuing full-body acupuncture with a licensed acupuncturist. This approach creates a win-win for all parties involved, patients, and providers. 

#### 3.3.1. Pueblo Community Health Center—Pueblo, Colorado

The Pueblo Community Health Center (PCHC) is a Federally Qualified Health Center (FQHC) that provided primary healthcare to over 24,000 patients in Pueblo, Colorado, in 2016. In addition to providing primary health and dental care they also integrate behavioral healthcare services and employ 17 licensed behavioral health providers including psychologists, social workers, and professional counselors. Of these, eight are ADSes. They utilize acudetox in individual sessions and in group settings. They provide six acudetox groups a week, four days per week—Monday–Thursday. One therapist reported that the patients will attend relaxation groups with acudetox more than any other group they offer. The patients report “a lot of internal healing without a lot of talking”. The best attendance is in a group that combines acudetox with coping skills—10 weeks of guided meditation and mindfulness training. The patients report that they like the combination of acudetox with mindfulness. One patient stated that his purpose in attending the group was, “I need to clear my soul”.

PCHC also introduced acudetox to an educational group for patients with diabetes. They are collecting data to see if the NADA protocol helps patients better deal with troubling emotions that come up for people with a diabetes diagnosis. The program tracks a patient’s weight, hemoglobin A1C, and blood pressure; they are also using the Problem Areas in Diabetes (PAID) scale to track feelings and emotions. 

The eight physicians and 20 midlevel providers were initially skeptical about the potential benefits of acudetox. Since implementation they have become enthusiastic about it as a crisis intervention tool and frequently request the behavioral health specialists to give a NADA treatment to the patients they are seeing. Examples of this include a patient who presented with such high blood pressure that transport to the emergency room was imminent. However, an acudetox treatment brought his blood pressure down 20–30 points within 30 min. This aspect of lowering blood pressure proves also helpful with obstetric patients. PCHC has recently started a medication assisted treatment program using buprenorphine for their pregnant clients with an addiction to opiates. Acudetox helps with the medication induction and then maintenance, keeping the women engaged in treatment. 

PCHC ADSes also use Vaccaria seeds (annual plant that grows in China—cow soapwort plant) applied with pieces of tape as acupressure on the Shen Men point in the ear either after a NADA treatment, for those who do not want needles, or for babies and children. One therapist related her experience with two babies born dependent on opiates and experiencing symptoms of neonatal abstinence syndrome. In both cases, the babies at three months were underweight and still struggling with agitation, irritability, and tremors. The therapist applied Vaccaria seeds bilaterally on the Shen Men point of the babies’ ears and taught the mothers how to do this with some massage of the points. Both babies went right to sleep. The mothers were given a supply of seeds to take home with them. 

Staff at PCHC frequently request an acudetox treatment when they feel stressed so this has been helpful in their staff wellness program. One staff member finds the treatment helps decrease the frequency and intensity of her migraine headaches. Now staff is considering a pain management group using acudetox because many patients have been able to decrease their medications for anxiety and pain when they get regular acudetox treatments. In their recent Health Resources and Services Administration (HRSA) 2017 survey PCHC was given a “best practice” for their use of acudetox. 

#### 3.3.2. Marillac Clinic—Grand Junction, Colorado

The Marillac Clinic is a Federally Qualified Health Center (FQHC) providing medical, integrated behavioral health, optical, and dental services to over 9000 underserved and low-income patients and their families at five clinic sites in Mesa County, Colorado. They have introduced the use of NADA acudetox since 2014 with great success. The medical providers refer patients to the social worker and ADS provider for help with smoking cessation, anxiety, relapse prevention, medication tapers and chronic pain. The ADS provider reports doing an average of 10–15 treatments per week with many more brief interventions using seeds or beads for acupressure. Using questionnaires he has found that 80% of the patients report reductions in chronic pain, stress, depression and anxiety, fear, and substance cravings with the use of acudetox as well as improvement in self-esteem and clear thinking. In May 2015, Marillac received a HRSA evaluation “best practice” for their use of the NADA protocol, which they refer to as “acu-wellness”. 

#### 3.3.3. St. Mary Corwin Hospital—Pueblo, Colorado

St. Mary Corwin Medical Center is a regional full-service, 408-bed hospital in Pueblo, Colorado, part of the Centura Health Network. Four social workers who work as psychiatric liaisons in the emergency department have been trained as ADSes. Patients who present in opiate withdrawal have been offered acudetox rather than the opiates they requested. Such patients have experienced some relief of withdrawal symptoms and are then referred to other acudetox providers or to the hospital’s own free weekly acudetox group, which routinely serves 20–30 people from the community at large.

A NADA-trained medical provider at the hospital successfully used acudetox with an uncooperative adolescent post overdose/suicide attempt. She said the ear acupuncture gave her a sense of well-being and she became more compliant. The provider reported that his primary goal in the use of the protocol in this case was to give her a sense of empowerment; to offer her a therapy that she could refuse without repercussion, and from that build some trust so that they could interact. He reported “it is definitely a nice arrow to have in the quiver when dealing with this population”. 

#### 3.3.4. Southern Colorado Harm Reduction Association—Pueblo, Colorado

In an effort to aid in the serious opioid epidemic happening in Pueblo, this non-profit organization opened a second needle exchange facility in July 2017, adding to the initial one that opened in July 2014. Needle exchange services are available once a week, seeing as many as 92 people in a day. Recently one of the staff members became trained as an ADS and started offering acudetox. Addressing the positive response she is seeing to the acudetox, she commented, “I believe we are prepping people for treatment”. The clients express sincere gratitude for the treatment. One reported that he came back because after the first session he “slept better than I have my entire life—I slept 12 h”. 

## 4. Discussion

Collectively, these results illustrate the importance of ADS provision. Our comparison of states provided a snapshot of the differences between a state that does not allow NADA-trained health care practitioners who are not also licensed acupuncturists or physicians to practice, to one that allows ADSes but only under strict onsite supervision, with one that allows behavioral health professionals to incorporate the NADA protocol broadly. The contrast in availability of NADA to clients is significant. Looking nationally at states, the results in Table 1 strongly suggest that where there is real potential for ADS integration, there can be more acupuncture available. Currently the only national information available is limited to substance abuse treatment programs. Future research could utilize a survey that captured actual integrated provision of acudetox within the entire behavioral health system. 

The interview-derived anecdotal Colorado reports highlight the creative applications of the NADA protocol within integrated behavioral healthcare settings facilitated by a change in the legislation. We want to note that Colorado acupuncturists as represented by their state association as well as acupuncture schools came to support the legislation over time. The Colorado examples highlight the kind of benefit that acudetox can bring to healthcare when in the hands of the frontline healthcare staff. Even though Colorado has 1513 licensed acupuncturists, integration in behavioral health settings could not have happened without ADS-friendly legislation. In comparison, California with 17,959 licensed acupuncturists but only 41 current ADSes has a lower percentage (only 8%) of treatment programs utilizing acupuncture as compared to Colorado (9.8%). 

The state with the highest number of ADSes in the NADA database is Michigan, where ADSes outnumber LAc, and almost 13% of the programs report using acupuncture. In fact, most of the top ten states providing some form of acupuncture in their treatment programs are ADS-friendly states. These include Connecticut (13.8%), New Mexico (16.2%), and Virginia (17%), all of whom have had ADS-friendly laws for more than 10 years, and in 2016 Connecticut expanded its law to all behavioral health settings. 

Limitations to this study include our reliance on extant data bases that offer only limited information. The survey we conducted was subjectively submitted to three states. The response rate was fairly low and the survey had no objective verification of responses. A more thorough survey would be expensive and difficult to pursue outside of a research institution or government agency. We are missing data from a few state boards that did not respond and states without acupuncture legislation may have acupuncturists that are not reported above. Some acupuncturists are licensed in several states. SAMHSA data only reflects addiction treatment programs, not other forms of behavioral health provision and as discussed above, the survey asks about “acupuncture”, not specifying NADA. The number of current ADSes reflects only those who are current members of NADA which may under-report NADA-trained persons who are not current members of the organization but who still practice acudetox. Furthermore, the ADS stipulation here does not differentiate between persons trained so the number would include licensed acupuncturists who are NADA-trained and current NADA members. 

As more states adopt ADS-friendly regulation that allows behavioral health professionals to use ear acupuncture for treating a wide range of behavioral health issues, the authors expect to amass similar case and program reports. It is our hope that more states, provinces, and countries will adopt rules and regulations that allow this practice because the need is great and urgent. It is also our hope that states with existing ADS regulations will expand scope of practice, and decrease supervision and training requirements to increase access.

In addressing social determinants of health, it is often noted that the providers need to reflect the population that they serve in terms of race and ethnicity. The U.S. national acupuncture community tends to be largely white or Asian (76.9% White/Caucasian, 16.6% Asian), according to a 2013 acupuncture job survey, whereas the larger community of behavioral health practitioners more commonly reflects their treatment communities [22]. That same job study reports that more than half of acupuncturists say they work in private practice. Full-body acupuncturists also tend to be clustered in major metropolitan areas and are not generally extensively trained in allopathic addiction or behavioral health treatment modalities. Conversely, behavioral health needs are everywhere and optimum treatment requires training in behavioral health. The NADA protocol is a tool that the behavioral health provider can add to their tool box. 

## 5. Conclusions

The practice of the NADA protocol by behavioral healthcare professionals facilitates greater access to this treatment for clients. Legislation that allows for non-acupuncturist ADSes to perform the NADA protocol supports this expansion. The NADA organization will continue to advocate for legislative changes that support the widespread application of acudetox within the healthcare and other relevant delivery systems such as disaster-response and community wellness initiatives. 

## Figures and Tables

**Figure 2:**
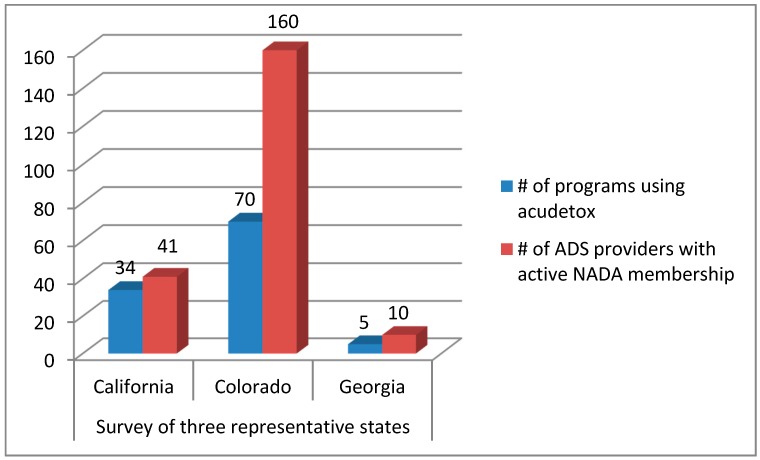
Survey of Programs Using Acudetox in Representative States.

**Table 1:** State-by-State Compilation of Data Ranked by Auricular Detoxification Specialist (ADS)-friendly Legislation and Ordered by Percentage of Acupuncture-reporting Substance Abuse Programs.

State	% Acu ^1^	SA Programs	ADS ^2^	LAc ^3^	ADS Rank	Limitations to ADS
Virginia	17	229	94	507	1	
Connecticut	13.8	224	91	400	1	
Michigan	12.7	479	178	171	1	
Wyoming	10.3	58	17	Unavailable ^4^	1	
**Colorado**	**9.8**	**399**	**160**	**1513**	**1**	
New Mexico	16.2	154	32	737	2	Supervision & Training
Rhode Island	11.5	52	5	166	2	Addiction only
Maryland	11.2	402	54	1138	2	Addiction only
Vermont	8.7	34	9	192	2	Addiction only
Arizona	8.1	46	51	35	2	Board-approved programs only
Tennessee	6.6	1430	48	217	2	Addiction only, Supervision
Washington	5.6	227	47	1550	2	Nurses, Physician Delegation
New Hampshire	4.7	60	5	133	2	New legislation
Texas	4.7	64	82	1265	2	Addiction only
Louisiana	4.5	488	13	55	2	Supervision
Missouri	3.5	80	9	132	2	Supervision
Indiana	3.4	268	36	115	2	Addiction only
New York	2.5	229	78	4398	2	Addiction only
Ohio	1.5	64	15	249	2	Nurses, Physician Delegation
Wisconsin	1.4	136	8	545	2	Physician Delegation
North Carolina	1.2	280	17	586	2	NP and PA, Physician Delegation
Arkansas	0.9	489	1	32	2	Addiction only
Delaware	0	201	18	7	2	High fees
Florida	11.2	716	22	2452	3	LAcs and Physicians Only
Oregon	10.3	223	38	1481	3	LAcs and Physicians Only
**California**	**8**	**358**	**41**	**17,959**	**3**	**LAcs and Physicians Only**
North Dakota Minnesota	5 4.1	428 157	9 7	See note ^5^ 606	3 3	LAcs and Physicians Only LAcs and Physicians Only
Hawaii	4	370	17	702	3	LAcs Only
Nevada	3.8	174	1	61	3	LAcs and Physicians Only
Massachusetts	3.1	265	13	1095	3	LAcs and Physicians Only
New Jersey	3	355	11	1000	3	LAcs and Physicians Only
Utah	3	371	2	167	3	LAcs and Physicians Only
Illinois	2.8	235	20	813	3	LAcs and Physicians Only
Pennsylvania	2.7	675	15	711	3	LAcs and Physicians Only
Maine	2.6	528	17	171	3	LAcs and Physicians Only
Iowa	2.5	922	0	66	3	LAcs and Physicians Only
Alaska	2.1	163	2	118	3	LAcs and Physicians Only
South Carolina	1.8	84	7	158	3	Direct Supervision
South Dakota	1.6	114	18	Unavailable ^4^	3	Not regulated
**Georgia**	**1.6**	**62**	**10**	**No response ^4^**	**3**	**Direct Supervision**
Montana	1.6	314	2	160	3	LAcs and Physicians Only
Nebraska	1.5	406	4	32	3	LAcs and Physicians Only
West Virginia	0.9	113	7	43	3	LAcs and Physicians Only
Kentucky	0.8	106	2	87	3	LAcs and Physicians Only
Alabama	0.7	363	8	N/A	3	Physician only
Idaho	0.7	136	1	157	3	LAcs and Physicians Only
Oklahoma	0.5	143	7	Unavailable ^4^	3	Not regulated
Kansas	0.5	204	2	Unavailable ^4^	3	New legislation
Mississippi	0	47	2	11	3	LAcs and Physicians Only

^1^ Percentage of Substance Abuse (SA) programs that reported offering acupuncture; ^2^ NADA-trained persons who are current members; ^3^ licensed acupuncturists as reported by individual state boards; ^4^ “Unavailable” indicates states which due to having no regulation or new regulation do not have numbers available and “No response” indicates state boards which did not respond despite numerous requests for information; ^5^ the board said it was unable to provide numbers.
